# Cells of the Immune System in Cardiac Remodeling: Main Players in Resolution of Inflammation and Repair After Myocardial Infarction

**DOI:** 10.3389/fimmu.2021.664457

**Published:** 2021-04-02

**Authors:** Irina Kologrivova, Marina Shtatolkina, Tatiana Suslova, Vyacheslav Ryabov

**Affiliations:** ^1^ Department of Clinical Laboratory Diagnostics, Cardiology Research Institute, Tomsk National Research Medical Centre, Russian Academy of Sciences, Tomsk, Russia; ^2^ Department of Emergency Cardiology, Cardiology Research Institute, Tomsk National Research Medical Centre, Russian Academy of Sciences, Tomsk, Russia; ^3^ Division of Cardiology, Department of Professional Development and Retraining, Siberian State Medical University, Tomsk, Russia

**Keywords:** myocardial infarction, myocardial remodeling, inflammation, resolution, immune cells, cellular heterogeneity, immune metabolism

## Abstract

The burden of heart failure (HF), developing after myocardial infarction MI, still represents a major issue in clinical practice. Failure of appropriate resolution of inflammation during post-myocardial injury is associated with unsuccessful left ventricular remodeling and underlies HF pathogenesis. Cells of the immune system have been shown to mediate both protective and damaging effects in heart remodeling. This ambiguity of the role of the immune system and inconsistent results of the recent clinical trials question the benefits of anti-inflammatory therapies during acute MI. The present review will summarize knowledge of the roles that different cells of the immune system play in the process of post-infarct cardiac healing. Data on the phenotype, active molecules and functions of the immune cells, based on the results of both experimental and clinical studies, will be provided. For some cellular subsets, such as macrophages, neutrophils, dendritic cells and lymphocytes, an anti-inflammatory activity has been attributed to the specific subpopulations. Activity of other cells, such as eosinophils, mast cells, natural killer (NK) cells and NKT cells has been shown to be highly dependent of the signals created by micro-environment. Also, new approaches for classification of cellular phenotypes based on the single-cell RNA sequencing allow better understanding of the phenotype of the cells involved in resolution of inflammation. Possible perspectives of immune-mediated therapy for AMI patients are discussed in the conclusion. We also outline unresolved questions that need to be solved in order to implement the current knowledge on the role of the immune cells in post-MI tissue repair into practice.

## Introduction

Study of treatment and prevention of atherosclerosis complications represents one of the most cutting-edge problems in the modern world. Despite the fact that modern interventional and pharmacological approaches allowed to greatly reduce mortality and morbidity after myocardial infarction (MI), patients remain at the high risk for the recurrent events in both short- and long-term (1). Even more so, the burden of heart failure (HF), developing after MI, still represents a major issue in clinical practice, especially HF with preserved ejection fraction (HFpEF) holding a high frequency in the population with no trends to decline (2, 3).

Left ventricular remodeling is the underlying basis of HF pathogenesis in patients after acute MI (AMI). The term “remodeling” was coined by N. Sharp in the end of 70s of the 20th century to describe structural and geometrical changes after AMI. Later on, it became more general term. Nowadays, heart remodeling means the complex process of disturbances in myocardial structure and function in response to the damaging myocardial overload and loss of the functioning myocardium (4).

Restoration of the myocardial blood flow with thrombolytic therapy and primary percutaneous coronary intervention, being an obligatory step in myocardial treatment, increases tissue deterioration even further and leads to ischemia/reperfusion myocardial injury (5). Deeper understanding of tissue remodeling post-MI may be the basis of the successful management of cardiac reparative processes.

Nowadays approaches to influence cardiac healing in the clinical practice are absent. Meanwhile state-of-the-art data emphasize an important role of immune system in orchestration of reparation and post-infarction tissue remodeling after AMI (6, 7). It determines both the degree of myocardial injury and the subsequent course of the disease. On the one hand inflammatory changes may favor death of cardiomyocytes and infarction expansion. But on the other hand, angiogenic and regenerative immune reactions may benefit resolution of inflammation and regeneration of injured myocardium. Immune cells remove necrotic cell debris and create milieu necessary for migration, proliferation, differentiation of fibroblasts and endothelial cells, which is required for reconstruction of extracellular matrix, neo-vascularization, scar formation and, hence, heart recovery (8).

Understanding of the key processes in the immune system during AMI may allow to create new diagnostic and therapeutic approaches to predict and prevent further HF development. Unfortunately, knowledge of the immune mechanisms during heart remodeling is incomplete and even clinical trials sometimes show contradictory results. Partially this may be explained by the fact, that many cells, which originally were considered to be proinflammatory and harmful by default, also may display powerful healing properties. The current therapeutic approaches are missing techniques which would have not only suppressed inflammation, but rather targeted its resolution. In the present review we will summarize knowledge of the roles that different cells of the immune system play in the process of post-MI inflammation development and resolution and the subsequent cardiac remodeling. In the end we will outline possible perspectives of immune-mediated therapy for AMI patients and outline pending questions concerning implementation of the current knowledge into practice.

## Pathophysiology of Post-Infarction Heart Healing

Cardiac repair after AMI consists of several phases: early inflammatory phase (first 72 hours after AMI), late reparative and proliferative phase (days 7-10), which are followed by maturation phase (9, 10).

The initial processes activated in the early phase are related to the maintenance of the cardiac output and normalization of the left ventricular wall strain. Left ventricular radius of curvature changes, which determinates different wall stiffness and distribution of the intraventricular volume. “Stretching” and thinning of the myocardium in the injured zone takes place, followed by inflammation and resorption of necrotic tissue. This ultimately leads to the so-called “myocardial infarct expansion”. Release of the intracellular content and destruction of extracellular matrix during inflammatory phase are associated with generation of danger-associated molecular patterns (DAMPs), non-specific stereotypic signals, which when recognized by pattern-recognition receptors (PRRs), lead to activation of the immune cells (10). The ultimate result of the inflammatory phase is degradation of extracellular matrix and phagocytosis of dead cells, also known as efferocytosis if it is associated with sensing of the ‘eat-me’ signals on apoptotic cells, such as phosphatidylserine (11).

Inflammatory phase should be followed by the reparative and proliferative phases to provide a wound healing and a proper scar formation. Intact myocardium is actively involved in the processes of the late remodeling. The aim of scar is prevention of myocardial rupture and partial limitation of the functional deterioration. Anti-inflammatory signaling, depletion of inflammatory neutrophils, fibroblasts proliferation and deposition of granulation tissue should be switched on to achieve this (12).

The final phase is maturation, associated with remodeling of extracellular matrix and lasting several months. This phase depends on the events that had occurred in the previous phases and is critical for the restoration of the heart function. Unsuccessful maturation may lead to the increase of myocardial stiffness, diastolic dysfunction and development of HF (10).

Cells of the immune system are present in myocardium at all the stages of heart repair. But the original dogma of their exclusively deteriorating role has been significantly shattered in the last decades. Many cellular populations have been shown to encompass both inflammatory and anti-inflammatory subsets, while properties of others have been shown to depend on the signals received from microenvironment. Timely switching from inflammatory to anti-inflammatory activity of the immune systems is crucial during post-myocardial healing. Meer inhibition of inflammation appeared to be harmful to the myocardial function and may even lead to the rupture of myocardium. On the contrary, adequate resolution of inflammation, defined as degradation of the inflammatory products, leading to cessation of inflammation and restoration of homeostasis (13), is essential to prevent the chronization of the inflammatory process. Key targets of inflammation resolution in AMI are limitation of infiltration of myocardial tissue with polymorphonuclear cells, decrease of the vascular permeability and proper clearance of the destroyed cells and components of extracellular matrix. Understanding the role which immune cells play in inflammation resolution will allow to elaborate appropriate diagnostic and therapeutic approaches to improve AMI outcomes.

## Specialized Pro-Resolving Lipid Mediators as Guides for Resolution of Inflammation in Injured Myocardium

Cellular traffic in the development of any inflammation is governed by the chemical mediators. Sterile inflammation developing in the course of acute MI is not an exception. The most potent mediators of inflammation are produced from the essential polyunsaturated fatty acids (PUFAs). Splenic leukocytes recruited to the myocardium represent an important source of these mediators, with macrophages being the major cellular subtype being involved (14). While acute phase of inflammation is primarily governed by prostaglandins and leukotrienes, recently identified specialized pro-resolving lipid mediators (SPM) participate in the resolution of inflammation (15). The timely switching of lipid metabolism from leukotriene B4 (LTB4) production to the synthesis of SPM limits attraction of pro-inflammatory leukocytes to the myocardium and provides efficient resolution of inflammation. SPM are classified into several subsets: lipoxins, resolvins, protectins and maresins. This classification is based on the nature of the precursor polyunsaturated fatty acid (16). SPM production becomes possible only in the course of intercellular interactions: formation of interactome between neutrophils and macrophages, neutrophils and platelets, neutrophils and eosinophils or endothelial cells provides the required sequence in the biosynthesis of lipid metabolites (17, 18). Synthesis of SPM leads to the termination of polymorphonuclear neutrophil (PMN) transmigration, alternative polarization of the macrophage phenotype and enhancement of efferocytosis of the apoptotic neutrophils (19). Even more so, these SPM have the capacity to modulate the functioning of the adaptive immune system, skewing activation towards regulatory T-cells’ subsets (20).

Myocardial infarction was associated with redistribution of leukocytes from the spleen to the heart tissue at day 1 together with the increased SPM generation in myocardium (21). STEMI patients presented with increase of SPM plasma levels already in the first hours after infarction, even before reaching the peak of high-sensitive troponin T (hsTnT), highlighting that processes of inflammation resolution are initiated in unison with its development (16). Production of pro-resolving mediators during MI possessed race- and sex-dependent signature patterns: black patients were characterized by the lowest level of resolving E1, while white males had the lowest production of protectin D1 (22). Administration of resolvin E1 (RvE1) to rats after ischemia/reperfusion limited the infarct size in a dose-dependent manner, while RvE1 treatment of cardiomyocytes *in vitro* significantly increased their viability and limited the rate of apoptosis (23). Injection of resolving D1 (RvD1) also improved heart function after the coronary artery ligation in mice and prompted synthesis of the internal RvD1, RvD2, maresin 1 (MaR1) and lipoxin A4 (LXA4) in the murine spleens (24). Of note, RvE1 administration during days 7-14 post-MI, on the contrary, adversely affected the myocardial function, due to the impaired recruitment of the monocytes/macrophages and reduced neovascularization (25). Lipoxins produced from arachidonic acid have been shown to upregulate the production of inflammatory cytokines IL-1β and IL-6 at day 1 post-MI (14).

Artificially induced impairment of the SPM signaling was associated with failure of inflammation resolution and dysfunction of leukocytes in the model of post-myocardial decompensated heart failure (26).

Besides lipid mediators the traffic of immune cells post-MI is also guided by other bioactive substances including annexin A1 (27), antimicrobial peptides (28), acute-phase proteins (29), and, obviously, various cytokines and chemokines (30). This creates a complex cross-talk between cells of the immune system, which, when unperturbed by aggravating factors, leads to the successful resolution of inflammation and restoration of myocardial function. Each cellular subset makes its unique input in the scenario of the development/resolution of inflammation following myocardial infarction.

## Neutrophils

Neutrophils (or polymorphonuclear neutrophils) represent the most abundant subpopulation of polymorphonuclear cells in the blood and are the first cells of the immune system arriving to the site of infraction (31). In humans their phenotype is described as CD11b+CD16+CD62L+ (CD11b+ Ly6G+F4/80 in mice) (32). They contain granules with proteases and biologically active substances. Granules are classified into azurophilic primary (contain cathepsin G, myeloperoxidase (MPO), defensins, neutrophilic elastase), specific secondary (contain NADPH oxidase, lysozyme, lactoferrin) and tertiary (contain metalloproteinases (MMP)).

Neutrophils are recruited to the infarcted myocardium during the first few hours, initiating local inflammation and tissue destruction (31). Of interest is that rather a large pool of the resident cardiac neutrophils has also been described, which primarily perform the surveillance role in the myocardium (33). The main function of the recruited neutrophils is to clear the site of myocardial injury from the necrotic tissue. Unfortunately this process is also associated with further tissue destruction. Neutrophilic activity is majorly mediated by phagocytosis, degranulation, production of reactive oxygen species and NETosis, recently described process, characterized by the release of chromatin fibers into extracellular space and formation of neutrophilic extracellular traps (NETs) (34). The neutrophilic role in post-infarct inflammatory response is so important that clearance of neutrophils from the myocardial tissue is considered to be one of the major signs of resolution of inflammation (35).

Elevated neutrophils are associated with bad prognosis in MI patients. The major detrimental effects are dependent upon the function of myeloperoxidase (MPO) secreted by neutrophils into the extracellular space (36). NETs released by neutrophils consist of chromatin, histones and granules with proteolytic enzymes. The intensity of NETs formation in the culprit lesion has been shown to directly correlate with the infarction size. Besides, NETs induced differentiation of monocytes into intermediate subset, associated with poorer prognosis (the role of monocytes will be discussed in the further chapter) (37). Crosslinking of NETs with plasma fibrinogen leads to thrombosis and “no-reflow” phenomenon (38).

However, neutrophils’ recruitment has been shown to be an obligatory requirement for the successful switching from inflammatory response to its resolution. This dual function of neutrophils may explain unsuccessful attempts of translation of anti-neutrophilic therapies into clinics (39). Apoptosis of neutrophils after degranulation represents a powerful stimulus to polarization of macrophages into anti-inflammatory phenotype, through the secretion of annexin A1, lipocalin, lactoferrin, cathelicidin and formation of apoptotic bodies (29). Release of these pro-resolving substances also suppresses further recruitment of polymorphonuclear cells in the infarct zone (32). Neutrophils may secrete annexin A1, lactoferrin and participate in chemokine scavenging, thus terminating transmigration of granulocytes to the infarcted myocardium (40). IFN-γ produced by natural killer (NK) cells and T-lymphocytes has been shown to be tightly involved in this process, stimulating resident macrophages to attract neutrophils to myocardial tissue (41). Prolonged lifespan of neutrophils under the influence of tumor necrosis factor (TNF) and IL-1β was associated with increased endothelial damage and adverse myocardial healing (42), while matrix metalloproteinase (MMP)-12 seems to favor neutrophilic apoptosis (17). Overall, MMP-12 has been shown to possess potent pro-resolving properties, influencing inflammatory signaling and controlling cellular clearance, and neutrophils appeared to be the source of MMP-12 themselves (43).

Switching between the production of inflammatory and pro-resolving lipid mediators also explains the dual role of neutrophils in the MI. Neutrophils are the main cells, expressing 5-lipoxigenase (LOX), which is responsible both for the synthesis of leukotriens and production of the various SPM initiated in the course of intercellular interactions in tissues (17, 18). As a result, neutrophils enhance the recruitment of other PMNs at the beginning of the development of inflammation, while suppress further PMN migration and facilitate efferocytosis in the resolution phase. We have encountered no studies describing the role of neutrophilic SPM in resolution of post-MI inflammation. However, lack or deficiency of 5-LOX was associated with the increased mortality of mice after chronic coronary ligation (44).

It has been shown that neutrophils undergo the continual change of the proteome in the first week after AMI, with Day 1 cells predominantly producing metalloproteinases, switching to upregulation of apoptosis and ECM reorganization phenotype at Day 3 and predominant expression of the reparative substances, including fibronectin, galectin-3, and fibrinogen at Day 7 (45).

Even more so, according to the recent data neutrophils may be subcategorized into N1- and N2-subpopulations, with CD206-N1-neutrophils being inflammatory and CD206+ N2-neutrophils – ant-inflammatory cells (31). In mice N1-neutrophils prevailed during the initial phase of infarction and N2-neutrophils peaked at days 5-7 after MI (31). In the model of chronic MI total depletion of neutrophils was associated with increased fibrosis and worsening of the heart function (29).

Current studies provided information that neutrophils are characterized by the even more significant heterogeneity than it was originally thought. In humans there were recognized CD177+, olfactomedin 4 (OLFM4)+, T cell receptor αβ+, CD49d+CXCR4+vascular endothelial growth factor (VEGFR)+ neutrophilic subsets. Neutrophils expressing programmed death-ligand (PD-L)1, arginase 1(ARG1), CD10 are reported to have potent immunosuppressive activity (46). However, it is still unclear, either newly identified neutrophilic phenotypes represent the true subsets, or rather refer to the definite functional state of neutrophils. Data concerning the functions of the recently identified subpopulations of neutrophils in myocardial infarction are presently absent.

## Eosinophils

Another subpopulation of granulocytes which role in myocardial infarction is usually overlooked is represented by eosinophils. In steady state they constitute 1-3% of leukocytes in peripheral blood and contain primary and secondary granules, as well as lipid bodies (47). Main active substances produced by eosinophils are galectin-10, major basic protein (MBP), eosinophil-derived neurotoxin (EDN), eosinophil cationic protein (ECP), eosinophil peroxidase (EPO) and derivatives of arachidonic acid. These substances are released in the course of degranulation, phagocytosis and formation of extracellular DNA traps (the process named EETosis), enabling eosinophils to kill extracellular parasites and participate in allergic reactions (47). Even more so, eosinophils are capable of presentation of antigens to Th2-lymphocytes and may even activate dendritic cells through the production of eosinophil peroxidase (EPO) (48). Eosinophils were also shown to be involved in the pathogenesis of myocardial infarction, reaching the peak in plasma by days 2-3 post-AMI (49). They may increase damage of myocardial tissue through the production of ROS by eosinophil peroxidase or induction of cellular cytotoxicity by granules’ contents or antibody-mediated cellular cytotoxicity (47).

Meanwhile, patients with low eosinophilic cell count were characterized by the bigger infarct size and higher rate of cardiac events during long-term follow-up (50). Thus, eosinophils have also been shown to mediate anti-inflammatory effects and input resolution of inflammation. Even though until recently they were regarded as a simple short-lived post-mitotic cellular subpopulation, nowadays a potent regulatory potential of eosinophils has been widely appreciated. Eosinophils possess a potential to produce variable growth factors, including TGF-α and -β, fibroblast growth factor (FGF), epidermal growth factor (EGF), platelet-derived growth factor (PDGF) and vascular-endothelial growth factor (VEGF), participating in angiogenesis and myocardial repair (51). However, balance between production of eosinophilic IL-5 and growth factors is pertinent, as mice overexpressing IL-5 were characterized by the insufficient production of ECM components and impaired wound healing (52). Eosinophils have also been confirmed to be the main source of protectin D1 and a range of resolvins, including novel resolvin RvE3, limiting infiltration of tissue with neutrophils (13).

Eosinophils also secrete IL-4, cytokine playing an essential role in differentiation of macrophages towards M2 phenotype, and regulating myocardial tissue regeneration (53, 54). Eosinophilic cationic protein mEar1 together with IL-4 was able to inhibit apoptosis is cardiomyocytes and prevent adhesion of neutrophils to endothelial wall, improving cardiac dysfunction (49). Most likely production of IL-4 is a key feature providing beneficial effects of eosinophils during AMI, as injection of the recombinant IL-4 to eosinophil-deficient mice rescued adverse remodeling of the left ventricle after the permanent ligation of the left coronary artery (54). However, the potency of treatment with IL-4 appeared to be time-dependent: administration of IL-4 lost its efficiency when injected at the advanced stages of the post-MI remodeling (55). Thus, time frame of the intercellular interactions has been shown to be critical for the successful myocardial healing following infarction. These findings highlight the importance of the proper “treatment window” choice when elaborating therapeutic approaches to manipulate inflammation and immune cells’ kinetics in AMI.

Distinct regulatory subpopulation of murine Siglec-F^int^CD62L+CD101^lo^ eosinophils (rEos, corresponding to human Siglec-8+CD62L+IL-3R^lo^ cells) with homeostatic functions has recently been described in lungs (48). They were also detected in gastro-intestinal tract, Peyer’s patches, adipose tissue, thymus and uterus, but there are currently no data on whether these cells exist in humans’ heart. Aforementioned rEos produce IL-4 in high quantities (48) and represent the cellular subset that potentially may be involved in resolution of post-infarct inflammation.

There still remains many unresolved questions in the role of eosinophils in post-infarct inflammation, such as gender bias, as many aspects of homeostatic and anti-inflammatory activity of eosinophils have only been confirmed in males, and were not established in females yet (49).

## Monocytes/Macrophages

The role of monocytes and macrophages in post-infarct inflammation has been described the most explicitly. Myocardium contains resident macrophages (about 7-8% of all non-cardiomyocytes), arising either from the yolk sac or fetal liver, and providing immune surveillance and regulation of cardiac functions (12, 56). Even though in steady state resident macrophages may proliferate to maintain their pool, they are rapidly depleted after myocardial injury (57). It is circulating monocytes that are going to be recruited and subsequently differentiate into macrophages to mediate development and resolution of inflammation. Myocardial infarction already in the first 30 minutes is characterized by the significant mobilization and influx of monocytes to the myocardial tissue, first from the patrolling pool in the peripheral blood and then from the splenic reservoir through the signaling *via* angiotensin II Type 1a receptor (58). These initially recruited monocytes may even be regarded as one of the stimuli that further attract neutrophils to the infarcted myocardium, as depletion of CCR2+ monocytes and macrophages was associated with impaired neutrophils extravasation during ischemia reperfusion injury in mice (59).

Several subsets of monocytes have been described based on their phenotype, however there is no consistency in the understanding of the further fate of these monocytic subpopulations. In men classification is based on the expression of molecules CD14 (lipopolysaccharide-receptor) and CD16 (low-affinity Fcγ-receptor). Classical monocytes in humans are described as CD14++CD16^neg^; in mice – Ly-6C^hi^. They constitute about 90% of all circulating monocytes and are characterized by high expression of CD62 and CCR2, which enables their recruitment to the site of injury towards the gradient of MCP-1 and chemokines CCL2 and CCL7. Classical monocytes produce inflammatory cytokines and nitric oxide and are able to differentiate into macrophages and dendritic cells. They peak at Day 3 after MI, and are involved in breakdown of necrotic myocardium (12). Even though there is no direct evidence that classical monocytes are precursors to macrophages with a certain phenotype, they are often called “inflammatory monocytes” (35). Blockage of CCR2 in mice was associated with increase of left ventricular ejection fraction (LVEF) and decrease of left ventricular end-diastolic volume (LVEDV) (60). Increased levels of classical monocytes were associated with impaired left ventricular functions six months after AMI (61). Meanwhile, the total depletion of monocytes and infiltrating macrophages during the 1^st^ week following myocardial injury in mice was associated with enhanced heart rupture and increased mortality (62).

Human non-classical monocytes are CD14+CD16^hi^ and murine non-classical monocytes are Ly-6C^lo^. High expression of chemokine receptor CX3CR1 is typical for non-classical monocytes, allowing their homing to the infarction site by gradient of chemokine fractalkine. Their function and origin are less clear than classical monocytes. Mainly they represent a patrolling monocyte population, being found close to the vessel wall in the circulation and scavenging oxidized lipids, cellular debris and pathogens (58). According to our data the frequency of non-classical monocytes inversely correlates with the severity of atherosclerosis in patients with stable coronary artery disease (63). One may speculate that it is non-classical monocytes that are essential for the resolution of inflammation in AMI, as late recruitment of monocytes in the models of acute inflammation did not depend on the CCR2 expression (64). Even though there is no direct evidence of the subsequent differentiation of non-classical monocytes into M2-macrophages, loss of fractalkine receptors was associated with skewing of macrophage balance to inflammatory subset in epididymal white adipose tissue (65). It has been shown that transcription factor NR4A1 (also known as Nur77) plays role in re-differentiation of classical monocytes into non-classical subpopulation. Absence of Nr4a1 in mice was associated with impaired LV function after AMI (66). Non-classical monocytes reach the peak at Day 5 after MI, but classical monocytes still predominate in numbers through all the course of infarction progression (12). Non-classical monocytes may regulate scar formation, angiogenesis and myocardial healing, through the secretion of VEGF and TGF-β (67). Absence of increase of non-classical monocytes is an unfavorable sign which was associated with reduction of LVEF (68).

There may be also identified so called intermediate monocytes (CD14++CD16^hi^), absent in mice. Most likely these cells represent a transition step between classical and non-classical monocytes. Their function is described primarily as inflammatory (12, 58). Intermediate monocytes also express CCR2 and angiopoietin-2 receptor (TIE-2), and thus are involved in regulation of angiogenesis (56). Levels of intermediate monocytes in AMI patients correlated with troponin concentration (69).

Differentiation of monocytes to macrophages is accompanied by increase of CD68 and MHC II expression in humans and F4/80 in mice, and reduction of CD14 expression (12). Macrophages play dual role in resolution of inflammation: clearing the tissue from necrotic remnants on one hand, and secreting pro-resolving mediators on the other hand (35). Similarly to monocytes, macrophages are characterized by heterogeneity, with different subsets involved in resolution of inflammation unequally. The common and most widely used approach to classify macrophages depends on the stimuli used to polarize macrophages *in vitro*. Monocytes will differentiate towards M1 macrophages under the influence of granulocyte-macrophage colony-stimulating factor GM-CSF and towards M2 macrophages under the influence of macrophage colony-stimulating factor (M-CSF) also known as colony stimulating factor 1 (CSF-1). The following post-differentiation stimulation with either LPS/IFN-gamma/TNF-alpha or IL-4/IL-13 will respectively initiate transition of M1/M2 macrophages into the activated state (70, 71). In vivo it is cytokines, DAMPs and growth factors that play the role of the polarizing agents (35).

M1 macrophages are characterized by the inflammatory phenotype and produce cytokines such as IL-1β, IL-6 and TNF-α, while alternatively activated M2 macrophages are anti-inflammatory and may activate fibroblasts, induce cell proliferation, collagen deposition and angiogenesis (12, 70). M2 macrophages may be further subcategorized into M2a, M2b and M2c cells, where M2a and M2c macrophages primarily cooperate with cells of the adaptive immunity and M2b cells play the role in regulation of inflammation (72).

Various types of cell death have been identified in the infarcted myocardium, including necrosis, apoptosis, necroptosis, autophagy-related cell death, pyroptosis and ferroptosis (11). M2 macrophages have been shown to be tightly involved in efferocytosis – a process of engulfment of the apoptic bodies, essential in the resolution of inflammation after AMI (73). The main feature of efferocytosis is clearance of the dead cells in the “immunologically silent” manner, as production of inflammatory cytokines is suppressed by efferocytic mechanisms (74, 75). Instead, macrophages switch to the production of TGF-β, IL-10, lipoxins, resolvins and other SPM (75). The growth-arrest specific gene 6 (Gas6)/myeloid-epithelial-reproductive tyrosine kinase (MerTK) pathway is employed by macrophages to recognize phosphatidylserine (PtdSer) on the surface of the dead cells and proceed to efferocytosis (11). Deficiency of MerTK was associated with impairment of efferocytosis and increase of the infarct size after the coronary occlusion in mice (76). Expression of MerTK appeared to be tightly associated with the monocytic scavenger receptor CD36: mice lacking CD36 were characterized by the reduced expression of MerTK on macrophages. The double knock out of CD36 and MerTK led to the increased post-infarct myocardial rupture (77).

In our previous collaborative works it has been shown that stabilin-1+ M2 macrophages peaked in patients’ heart tissue in the regenerative phase of MI (days 4-10) and did not decrease further on, predominating in the infarct area (78). Specific targeting of genes responsible for M2 polarization was followed by the impairment of left ventricular function after coronary artery ligation in mice (79). Meanwhile, blocking of M1 polarization pathways led to the reduced LV dilation at 3 weeks post-coronary artery ligation (80).

However, M1-M2 paradigm still carries some inconsistency. Unexpected results were obtained by Yang M. et al. (81), who knocked out GATA3, a transcription factor that appeared to be involved in the development of M2-phenotype (81). This was associated with the prolonged persistence of CCR2+/Ly-6C^hi^ inflammatory macrophages in the post-infarct myocardial tissue. Yet, animals performed improved LV function in 2 months of observation compared to intact animals, thus, questioning the detrimental role of Ly-6C^hi^ macrophages in cardiac remodeling. It is worth noting that the decreased accumulation of neutrophils was also observed in animals after GATA3-knock out, thus representing the confounding issue of the study (81).

One of the alternatives is to avoid classification of macrophages into M1 and M2, but rather indicate the stimulus used to activate macrophages: M_LPS, M_IFN‐γ, M_IL‐4, M_IL‐10, M_IL‐6, etc. RNA expression profile and CD markers vary greatly between M_LPS and M_IFN‐γ (previously reported together as M1 subpopulation) and between M_IL‐4 and M_IL‐10 (previously reported together as M2 macrophages) (82). The use of single-cell RNA sequencing (scRNA-seq) allowed to identify as many as 11 clusters of mononuclear phagocytes with distinct gene expression profiles in the heart of naïve mice (57). There was observed predominantly inflammatory gene expression at day 3 post-coronary artery ligation, and reparative genes expression at day 7, accompanied by mixed expression of M1 and M2 marker-genes, further emphasizing over-simplification of macrophages categorization into M1 and M2 cells (83).

## Mast Cells

Mast cells represent cells of immune system of myeloid origin, residing in mucosa and connective tissue and mainly being involved in development of allergic conditions. They are abundant in human heart and coronary lesions and were shown to participate both in plaque rupture and processes of myocardial remodeling after infarction (84–87). Mast cells are able to produce various inflammatory cytokines including TNF-α, IFN-γ, IL-6, amines (mainly histamine) and proteases, regulating activity of matrix metalloproteases, collagen degradation and thus playing role in organization and resolution of inflammation and fibrosis in myocardium (87).

Phenotype of mast cells is characterized by expression of c-Kit, Fc-εRI and various mast-cell-specific proteases (84). Depending on the proteases contained in granules mast cells have been classified into two subsets: MC_T_ (with granules containing only tryptase) and MC_CT_ (with granules containing tryptase, chymase, cathepsin G, and carboxypeptidase). Heart mast cells have been described to have MCct phenotype (87). Biological activity of mast cells may be realized through degranulation and secretion of the granules’ content, secretion of cytokines or production of lipid mediators, depending on the nature of the stimuli received during activation (84). Main route of mast cells activation is represented by the cross-linking of FcεRI with IgE (85). Secretagogues of mast cells, mediating unspecific degranulation, include complement components, stem cell factor (SCF), defensins produced by neutrophils, substance P, neurotensin, endothelin-1, reactive oxygen species, IL-33 (ligand of ST2 receptor) (85, 87). Estrogen has been shown to down-regulate mast cell activity (87).

Mast cells have been found in media of infarct-related and infarct-unrelated arteries at different stages of infarction, and in myocardial tissue itself, implying that they may participate both in initiation and termination of the post-infarct inflammation (86). Treatment of isolated rat heart with ketotifen (mast cell membrane stabilizer) reduced ischemia/reperfusion injury (88). Retrospective and prospective studies investigating effects of histamine type 2 receptors (H2) antagonists showed reduction of brain-natriuretic peptide (BNP) production and decrease of the left ventricular dilation together with improvement of the heart failure in patients (89).

Despite evident inflammatory properties of mast cells, their main function during cardiac tissue remodeling remains to be associated with regulation of fibrous tissue metabolism (84). Mast cells may be both enhancers and inhibitors of post-myocardial fibrosis, reaching their pick at day 7 after MI. Pro-fibrotic properties are mediated primarily by chymase and tryptase, which are known to be activators of TGF-β and angiotensin II, the well-known promoters of fibroblasts activity. In addition, MC store basic fibroblast growth factor (bFGF) in their granules. MC also produce and secrete anti-fibrotic mediators such as IL-10, IL-13, CXCL-10 and VEGF-A (84).

Another input of MC in resolution of inflammation is mediated by activation of protease activated receptor (PAR)-2 on cardiomyocytes by tryptase. As a result of this activation anti-inflammatory 15-deoxy 12,14 prostaglandin J2 (15-d-PGJ2) is produced from Cyclooxygenase-2 (COX-2) (90). 15-d-PGJ2 regulates timely resolution of inflammation at the level of transcription, inhibiting proinflammatory factors NF-κB, STAT3, and activator protein 1 (AP1) and stimulating pro-resolving E2-related factor 2 (Nrf2). Besides, it inhibits protein translation, thus limiting the development of inflammation (91).

## Dendritic Cells

Dendritic cells (DCs) represent an extremely heterogeneous population of immune cells, both of myeloid and lymphoid origin. Their main function is presentation of antigens to T-lymphocytes. DCs express MHC II, similarly to monocytes and macrophages, but belong to a distinct cellular population (92). They also secrete various cytokines and growth factors, regulating immune response and inflammatory processes (92).

Two major DC subpopulations were identified in men and mice, such as plasmacytoid DCs (pDCs) and conventional DCs (cDCs), further classified into CD141+/CD103+ cDCs1 and CD1c/CD11b+ cDCs2 lineages (93). Both pDC and cDC subsets may be found in myocardium in steady state, as well as double-negative CD103-CD11b- pre-cDCs and poorly characterized population of monocyte-derived CD64+CD11c+MHCII+ DCs (moDCs) (94). Myocardial infarction was associated with the increase of сDCs and moDCs in the heart (95). But only cDCs2 increased expression of co-stimulatory molecule CD86 and migrated into the draining lymph nodes initiating activation of autoreactive IL-17+/IFN-γ+ T-lymphocytes (95).

DCs may modify the process of post-MI healing through activation of T-lymphocytes in draining lymph nodes, production of inflammatory cytokines and direct activation of fibroblasts (94). Besides antigen presentation to T-lymphocytes DCs may also influence the development of inflammation through the production of exosomes enriched in micro (mi)-RNA (96, 97). Infiltration of myocardium with DCs after left anterior descending coronary artery ligation in mice was characterized by spatial-temporal dynamics: DCs could be found in ventricle and septum at day 3, and appeared in the left atrium at day 7 (94).

Experimental works using depletion of DCs to study their function show inconsistent results. Depletion of conventional DCs in chimeric mice was associated with decrease of macrophages at day 7 post-MI, followed by reduction of infarct size, prevention of ventricular remodeling and improvement of cardiac function (94). However, in the series of other works DCs depletion was also associated with decrease of anti-inflammatory T-regulatory lymphocytes and impairment of the myocardial healing. Mixtures of cytokines IL-37 and troponin I led to the creation of tolerogenic DCs, which beneficially influenced the process of post-MI remodeling (98). Local injection of tolerogenic DCs induced activation of M2 macrophages through T-regulatory lymphocytes and favored resolution of inflammation, wound healing and ventricular systolic function (99). Biologically active molecules mediating pro-resolving functions of tolerogenic DCs include galectin-1 and pentraxin 3 (100, 101).

Thus, the ultimate result of the DCs activity in the course of AMI depends on the dendritic cells’ subpopulation being involved and subsequent type of the recruited effector cells. Ideally, activation of the conventional DCs should be followed by the increase of the tolerogenic DCs’ activity to provide timely resolution of inflammation.

## T-Lymphocytes

T-lymphocytes are the cells of the adaptive immunity. Presentation of the specific antigen by antigen-presenting cells in secondary lymphoid organs is an obligatory stage in their activation and clonal expansion (102). Population of T-lymphocytes may further be sub-divided into helper CD4+T-lymphocytes, secreting cytokines, and cytotoxic CD8+ T-lymphocytes, able to lyse pathologically changed cells directly (103). Each population may play either inflammatory or regulatory role (102).

Cells of the adaptive immunity attracted attention of the researchers not so long ago, and their function during MI remains elusive and stem from experimental works. It has been shown that RAG1 knock-out (KO) mice, which lacked T-lymphocytes, developed infarction of smaller sizes, compared to the wild-type animals. The primary role for modulation of the injury size has been ascribed to CD4+ T-lymphocytes, with interferon-γ (IFN-γ) and IL-17 being tightly involved in the process (104, 105). These cytokines amplify death of cardiac cells and stimulate proliferation of fibroblasts. Deficiency of IL-17A or γδ-T cells (main producers of IL-17 in the heart) alleviated left ventricular dysfunction after myocardial infarction (105). Meanwhile, decrease of Th2 lymphocytes was associated with increased severity of STEMI and high risk of adverse cardiovascular events (106), while administration of IL-4, the Th2 key-stone cytokine, had pro-resolving effects (55).

There is an opinion that activation of CD4+ T-lymphocytes during AMI is antigen-independent, associated with the recruitment and activation of effector memory T-cells through recognition of alarmins by TLR (107). Another pathway of T-lymphocytes’ activation is mediated by MMP-9 *via* cleavage of CD31, which normally inhibits signaling through T-cell receptor (TCR) (108).

At the same time, pro-inflammatory milieu, formed during MI, creates conditions favorable for activation of autoreactive clones of T-lymphocytes, which may trigger autoimmune destruction of the myocardial tissue (109). Heart-specific T-lymphocytes receive positive signal for differentiation while polyclonal inhibition of other T-lymphocytes’ clones is observed (110). Priming of T-cells takes place in mediastinal (heart-draining) lymph nodes, which enlarge after MI. Myosin heavy chain α (MYHCA), missing in thymus and not participating in negative selection of T clones, was shown to be the dominant epitope associated with activation of T-lymphocytes during MI (110).

T-lymphocytes are attracted to injured myocardium and are detained there. One of the possible chemokines, involved in this process, is fractalkine, recognized by T-lymphocytes’ receptor CX3CR1 (111). T-cells may also participate in microvascular-obstruction during ischemia-reperfusion injury, release inflammatory cytokines and increase cellular infiltration of the myocardial tissue even further ultimately leading to the aggravation of myocardial injury (111). This activation of CD4+ T-lymphocytes is a prerequisite process after MI, as absence of CD4+ cells in animals was associated with poorer outcome (102).

Cytotoxic CD8+ T-lymphocytes were also shown to be involved in post-infarction inflammation (112). CD8+ T-cells as well may have both beneficial and detrimental effects in myocardial infarction. Deficiency of cytotoxic T-cells was associated with better restoration of heart physiology, but scar formation was impaired. Mice deficient for CD8+ T-lymphocytes died post-MI due to the myocardial rupture caused by dysregulated fibrosis and increased inflammation (113).

Protective effects mediated by T-lymphocytes may be mediated by the distinct CD4+ and CD8+ subsets positive for expression of angiotensin receptor AT2 (114, 115). But primary protective function during MI is, of course, mediated by subpopulation of T-regulatory lymphocytes.

Tregs represent a distinct subpopulation of CD4+ T-lymphocytes, characterized by anti-inflammatory activity. Their major role in infarction is repair of myocardial tissue through suppression of inflammatory processes and activation of fibrosis (102). Natural Tregs appear in the infarction zone already in the first hours after MI and persist for at least 7 days (116). Treg recruitment most likely involves chemokine receptor CCR5 (117). Myosin-specific T-helper lymphocytes are attracted to the site of myocardial infarction and undergo *in situ* conversion to Tregs, which were characterized by enhanced expression of inhibitory check-point molecules, such as CTLA-4, TIGIT (T cell immunoreceptor with Ig and ITIM domains), PD-1, BTLA (B- and T-lymphocyte attenuator) (110).

Ectoenzymes CD39 and CD73 are expressed on the membrane of Tregs. Ectonucleoidase activity of these molecules input to the increase of the local adenosine concentration, which has been shown to suppress inflammatory activity of conventional CD4+ T-lymphocytes in myocardial injury (104, 118). Tregs can influence the differentiation of macrophages in the site of myocardial infarction, and direct it towards M2 phenotype, inducing expression of arginase-1, interleukin-13, osteopontin, and TGF-β (119). Disruption of this interaction was associated with failures in the formation of extracellular matrix in scar region and myocardial rupture (120). Crosstalk between Tregs and macrophages promoted efferocytosis in the models of acute inflammation (121). However no data is available on this effect of T-regulatory lymphocytes in AMI. Tregs may also improve cardiac tissue remodeling in a paracrine manner directly stimulating proliferation of cardiomyocytes through the secretion of six main factors (Cst7, Tnfsf11, Il33, Fgl2, Matn2, and Igf2) (116).

Statin intake before percutaneous interventions after MI was associated with the increase of Tregs among PBMC (122). Expansion of Tregs *via* agonistic antibodies led to the reduction of the recruitment of neutrophils, monocytes and lymphocytes to the site of myocardial injury and suppressed activation of CD8+ lymphocytes (123). Artificial depletion of Tregs was associated with unfavorable remodeling of the left ventricle, development of apical aneurisms and even cardiac ruptures (116, 120). This underscores an important role of Tregs in reparation of the injured myocardium.

## Myeloid-Derived Suppressor Cells

Myeloid-derived suppressor cells (MDSC) represent a recently described subpopulation of cells that originate from myeloid precursors in bone marrow, blocked at different steps of their development. This subpopulation is highly heterogeneous and at present includes polymorphonuclear MDSC (PMN-MDSC), monocytic (M-MDSC) and early-derived MDSC (eMDSC) (124). In steady state they may be found in bone marrow, but do not possess any suppressive activity. In the course of inflammatory disorders and tumorigenesis they appear in the secondary lymphoid organs and peripheral tissues (125). Role of these cells in the course of development of MI is not yet studied, but most likely they exhibit pro-resolving functions, suppressing T cell mediated immune responses (126).

Administration of rapamycin suppressed activation of the mammalian target of rapamycin (mTOR) and activated MDSC during acute kidney injury (127). This activation was accompanied by the decrease of CD4+ and CD8+ T-lymphocytes infiltration and decrease of inflammatory cytokines production (IL-1β, IL-6 and IFN-γ). Production of anti-inflammatory TGF-β increased on the contrary (127). Expression of indoleamine 2,3-dioxygenase (IDO) by MDSCs was associated with metabolic starvation of T cells and directed development of DCs towards tolerogenic subset (128). MDSC also exhibited anti-hypertrophic and anti-inflammatory effects when co-cultured with cardiomyocytes, through the secretion of IL-10 and nitric oxide (129).

MDSCs were elevated in patients with HF, and their depletion aggravated heart function (129). The kinetics of MDSCs’ recruitment to myocardial tissue and potential factors being involved in regulation still need to be elucidated.

## B-Lymphocytes

B-lymphocytes represent cells of the adaptive immunity, characterized by the production of antibodies and mediating humoral immune response. Two major types of B-lymphocytes have been described: B1-lymphocytes (functioning in T-independent manner; also called “innate-like” B-lymphocytes) and B2-lymphocytes (interaction with T-lymphocytes is required for proper function; also called conventional B lymphocytes) (130). B2 lymphocytes predominate in circulation. Surprisingly, B-lymphocytes are the most abundant lymphoid cells in myocardium, both in mice, and in men (131, 132). After myocardial infarction their numbers gradually increase nearly 5-fold. The time of reaching the peak values will depend on the presence of ischemia-reperfusion (day 7 – in non-reperfused myocardium; day 3 – after ischemia-reperfusion injury of the myocardium) (133).

The main effector molecules produced by B-lymphocytes are antibodies. Antibody production in performed by plasma cells – the terminal stage of B-lymphocyte differentiation. Antibodies increased destruction of myocardial tissue, as blocking of IgM led to the significant reduction in ischemia-reperfusion injury (134). At the same time, specialized pro-resolving mediators 17-hydroxydocosahexaenoic acid (17-HDHA) and resolvin D1 increased production of IgM and IgG from B-cells, suppressing secretion of B-derived IL-6 and IL-10 (135). Potentially, balanced antibody production may benefit resolution of inflammation through clearance of cellular debris.

Besides antibodies, B cells are also capable to secrete cytokines and chemokines. The production of CCL7, attracting Ly-6C^high^ monocytes to myocardium, has been shown to be dependent upon B-lymphocytes. Depletion of B2-lymphocytes from circulation by anti-BAFF of anti-CD20 antibodies disrupted monocytes recruitment and was associated with the reduction of infarct size and favorable heart remodeling (136).

Meanwhile, injection of B-lymphocytes into early post-infarcted myocardium reduced apoptosis in cardiomyocytes and preserved left ventricular function, while other cells of myeloid and lymphoid lineage as well as combinations of bone marrow cell subsets had no effect (137). Recently described population of IL-10 producing CD5+ “regulatory B-lymphocytes” may be the one to play anti-inflammatory function (138). However, an exact mechanism mediating this protecting effect of B-lymphocytes has not yet been described.

## NK Cells

Natural killer (NK) cells constitute 2-5% of all peripheral blood leukocytes and belong to the heterogeneous group of innate lymphoid cells (ILC), type I ILC in particular. They exhibit lymphoid cellular morphology, but do not have antigen specificity. Instead, they express a great variability of invariant receptors, recognizing both self and non-self antigens. NK activation depends on the balance between activation and inhibitory signals they receive during interaction with their target cells. NK cells express transcriptional factor T-bet and produce IFN-γ, perforin and granzyme B (139).

NKs’ role during MI still remains poorly characterized. NK cells appeared to be the major source of IFN-γ during angiotensin (AT) II induced vascular dysfunction (140). NK cells may interact with inflammatory macrophages through the cytokine axis IFN-γ/TNF-α/IL-12 whereby potentiating activity of one another and increasing inflammation in infarction zone (141).

At the same time there are data pointing to the possibility of insufficient NK function during MI. Patients with acute coronary syndrome were characterized by the lower numbers of NK cells in the peripheral blood compared to patients with stable angina (142). One of the possible explanations of the decreased NK numbers in CAD patients is high susceptibility of these cells to apoptosis induced by various stimuli (143). Observed NK deficiency during MI was associated with the reduced capacity to limit cardiac cell apoptosis and modulate development of fibrosis in post-infarcted myocardium (144). Thus, NK cells may also fulfil protective function in CAD patients. Most likely the balance between various stimuli will determine the ultimate role of the NK cells in infarct and peri-infarct zones.

## iNKT Cells

A distinct minor subpopulation of invariant natural killer T (iNKT) cells (0.01 – 0.5% of all peripheral blood leukocytes) simultaneously expresses NK activating and inhibitory receptors and an invariant αβ TCR which can recognize lipid and glycolipid antigens (mainly DAMPs) (145). Infiltration of activated iNKT into post-infarcted myocardium ameliorated left ventricular (LV) remodeling and development of heart failure after MI (145). Absolute numbers of circulating iNKT in AMI patients were decreased and could have been used to predict restenosis in long-term follow-up (146).

Mechanisms of iNKT protective effects during MI were not identified yet, but in the case of sterile inflammation in the liver iNKT have been shown to infiltrate the injured site and orchestrated healing *via* production of IL-4 and switching of macrophages to patrolling phenotype (147). Similar pattern of iNKT activity may exist in post-MI injury, but further research to prove this concept is required.

Glycosphingolipid α-galactosylceramide (αGC) is one of the most potent stimulators of both NK and iNKT activity. Administration of αGC to mice with ischemia/reperfusion reduced the infarction size, but whether this effect was mediated by iNKT cells or represents epiphenomenon remains disputable (148).

## Future Directions

**Figure 1:**
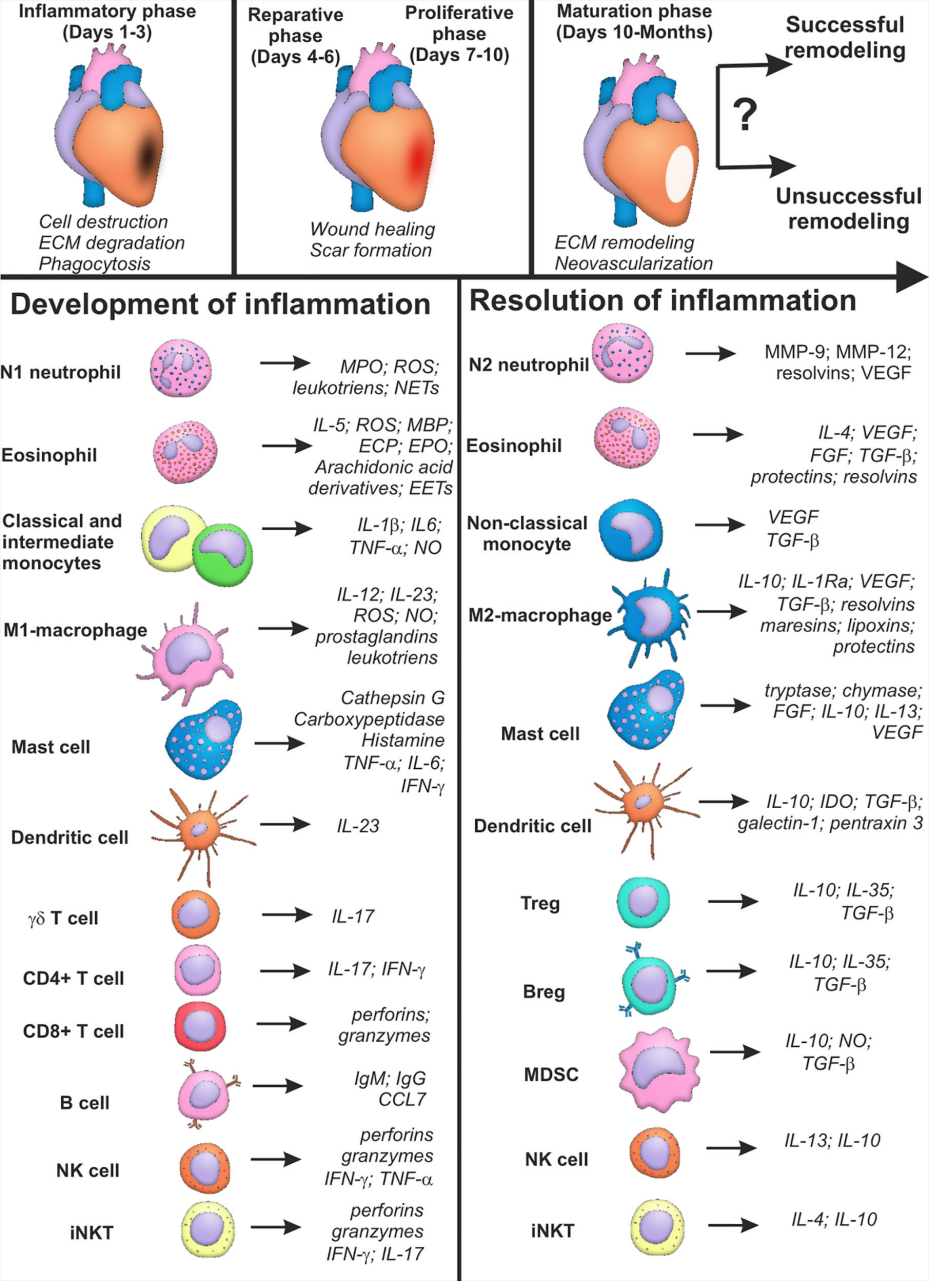
Summary characteristics of immune cells involved in development and resolution of inflammation post-myocardial infarction. Cells of the immune system may input either successful or unsuccessful myocardial remodeling after myocardial infarction, being involved in all the three phases of cardiac repair: inflammatory phase; reparative and proliferative phase; maturation phase. Some cellular populations (monocytes, macrophages, lymphocytes) are represented by two or more subsets with opposite or complementary effects. At the same time, some immune cells may exhibit either inflammatory or reparative properties, depending on the nature of the signal and cellular milieu (eosinophils, dendritic cells, mast cells, NK cells, iNKT cells). Possibility exists that new distinct subpopulations of immune cells are yet to be described, and inflammatory and reparative functions will further be ascribed to different cellular subsets. CCL CC, Chemokine ligand; CD, Cluster of differentiation; ECP, Eosinophil cationic protein; FGF, Fibroblast growth factor; IFN, Interferon; Ig, Immunoglobulin; IL, Interleukin; IL-1Ra, Interleukin-1 receptor antagonist; MBP, Major basic protein; MMP, Matrix metalloproteinase; MPO, Myeloperoxidase; NO, Nitric oxide; ROS, Reactive oxygen species; TGF, Transforming growth factor; TNF, Tumor necrosis factor; VEGF, Vascular endothelial growth factor; Question marks indicate absent or incomplete data and facts that have not been sufficiently studied for myocardial infarction.

Thus, cells of the immune system are unequivocally involved in development and resolution inflammation during myocardial infarction. The main features of the phenotypes, active molecules and effector activity of the immune cells during AMI are summarized in **Table 1**. The same cellular population may exhibit both inflammatory and pro-resolving properties (**Figure 1**), which, being combined with the excessive complexity of intercellular interactions, creates a well-tuned, but highly susceptible to perturbations, system of immune regulation in myocardial remodeling. This may explain unsuccessful attempts of remodeling modulation *via* targeting of the single signaling pathways or definite cellular populations (39). Besides, the majority of data concerning the role of immune cells in myocardial infarction have been obtained in experiment. Clinical context complicates existing regularities of immune cell functioning during AMI with such confounding variables as aging, comorbid conditions, variable genetic background, etc. (40).

**Table 1:** Summary characteristics of immune cells involved in development and resolution of inflammation in acute myocardial infarction.

Cell types	Development of inflammation				References	Resolution of inflammation				References
	Subpopulations	Surface Markers	Active molecules	Effector activity		Subpopulations	Surface Markers	Active molecules	Effector activity	
**Neutrophils**	N1-neutrophils	CD11b	MPO	NETosis	(31, 34, 36, 37)	N2-neutrophils	CD11b	VEGF	Macrophages polarization	(31, 43, 45, 149)
		CD16	ROS	Degranulation			CD206+	MMP-9	Angiogenesis	
		CD15	leukotrines	Phagocytosis			?	MMP-12		
		CD87								
						N1-neutrophils	CD11b	resolvins	SPM synthesis	(17, 18, 29, 31, 32, 40)
							CD16	annexin A1	Formation of apoptotic bodies	
							CD15	lipocalin		
							CD87	lactoferrin		
								cathelicidin		
**Eosinophils**	Inflammatory eosinophils?	Siglec-F^hi^ (mice)	ROS	EETosis	(47, 52)	Regulatory eosinophils?	Siglec-F^int^ (mice)	IL-4	Macrophages polarization	(13, 49, 51, 53, 54)
		CD62L–	major basic protein	Degranulation			Siglec-8+ (humans)	TGF-β	Angiogenesis	
		CD49d	eosinophil cationic protein	Oxidative burst			CD62L+	VEGF		
		CD101^hi^	eosinophil peroxidase				CD101^lo^	FGF		
			arachidonic acid derivatives				?	resolvin RvE3		
			IL-5					protectin D1		
**Monocytes**	Classical monocytes	CD14++CD16^neg^/Ly-6C^hi^ (mice)	TNF-α	Secretion of inflammatory cytokines	(12, 67)	Non-classical monocytes	CD14+CD16^hi^/Ly-6C^lo^	VEGF	Secretion of anti-inflammatory and angiogeneic cytokines	(12, 67)
	Intermediate monocytes	CD14++CD16^hi^	IL-1β				CXC3R1	TGF-β		
		CCR2	IL-6							
		TIE-2	NO							
**Macrophages**	M1-macrophages?	CD68	IL-12	Phagocytosis	(12, 70)	M2- macrophages (M2a, M2b, M2c)?	CD206	IL-10	Secretion of anti-inflammatory and angiogeneic cytokines	(72, 73, 75, 78)
		MHC II	IL-23	Secretion of inflammatory cytokines		CD163	IL-1Ra			
		CD86	ROS			Stabilin-1	TGF-β			
			NO				VEGF			
			leukotriens				Arginase			
							SPM (resolvins, maresins, lipoxins, protectins)	SPM synthesis		
								Efferocytosis		
**Mast cells**	MC_CT_	CD64	cathepsin G	Secretion of inflammatory cytokines	(84, 87)	?	?	chymase	Regulation of fibrosis	(84, 90, 91)
		CD117	carboxypeptidase	Degranulation				tryptase		
			histamine	Collagen degradation				bFGF		
			TNF-α					IL-10		
			IL-6					IL-13		
			IFN-γ					VEGF-A		
**Dendritic cells**	pDC	MHC II	IL-23	Antigen presentation to Th1/Th2/TH17	(92, 93, 95)	Tolerogenic DC	MHC IIlow	IDO	Antigen presentation to Treg	(98–101)
	cDC	CD80/CD86		Secretion of inflammatory cytokines			CD80/CD86low	IL-10	Inhibition of TCR signaling	
	moDC	CD1c/CD11b (mice)					PDL-1,-2	TGF-β		
		CD141/CD103 (mice)					CTLA-4	galectin-1		
								pentraxin 3		
**T-lymphocytes**	CD4+ T-helper	CD3	IFN-γ (in CD4+ Th)	Autoreactivity	(104, 105, 109, 111, 112)	Tregs	CTLA-4	TGF-β	Secretion of anti-inflammatory cytokines	(102, 104, 110, 114, 115, 118)
	CD8+ T-cytotoxic	CD4	IL-17 (in CD4+ Th and γδ T cells)	Secretion of inflammatory cytokines		AT2R+ T cells	PD-1	IL-10	Apoptosis of inflammatory cells	
	γδ T cells	CD8	perforins (in CD8+ Tc)	Cytotoxicity in cardiomyocytes?			BTLA	IL-35	Increase of extracellular adenosine	
		γδ TCR	granzymes (in CD8+ Tc)				CD39			
							CD73			
**Myeloid-derived suppressor cells**	?	?	?	?	?	PMN-MDSC	HLADR-CD14-CD15+CD11b+	IL-10	Immune suppression?	(124, 126–128)
						M-MDSC	CD33+CD14+CD11b+	NO		
						eMDSC	Lin-CD33+CD11b+	TGF-β		
								IDO		
								arginase 1		
**B-lymphocytes**	B1-lymphocytes	CD19	IgM	Antibody production	(134, 136)	regulatory B-lymphocytes	Tim-1	IL-10	Secretion of anti-inflammatory cytokines?	(138)
	B2-lymphocytes		IgG	Chemokines production				TGF-β?		
			CCL7					IL-35?		
**NK-cells**	?	CD69	IFN-γ	Secretion of inflammatory cytokines	(139–141)	?	Inhibitory receptors:	IL-13	Secretion of anti-inflammatory cytokines	(139, 143, 144)
		Activating receptors:	TNF-α	Recognition of DAMPs			KIR	IL-10	Recognition of “self”-molecules	
		NKp44	perforin	Cytolysis			NKG-2A		Prevention of cytolysis	
		NKG2D	granzyme							
		CD226								
**iNKT**	?	Activating receptors	IL-17	Secretion of inflammatory cytokines	(145)	?	Inhibitory receptors	IL-4	Secretion of anti-inflammatory cytokines	(147)
		Invariant αβ-TCR	IFN-γ	Recognition of DAMPs				IL-10	Recognition of “self”-molecules	
				Cytolysis					Prevention of cytolysis	

BTLA, B- and T-lymphocyte attenuator; CCL, CC chemokine ligand; CCR, C-C chemokine receptor; CD, cluster of differentiation; CTLA, cytotoxic T-lymphocyte-associated protein; DAMP, damage associated molecular pattern; DC, dendritic cells; IDO, indoleamine 2,3-dioxygenase; IFN, interferon; Ig, immunoglobulin; IL, interleukin; KIR, killer cell immunoglobulin-like receptor; Lin, lineage; MC, mast cell; MHC, major histocompatibility complex; MMP, matrix metalloproteinase; MPO, myeloperoxidase; NKG2D, natural killer group 2 member D; NO, nitric oxide; PDL, programmed death ligand; ROS, reactive oxygen species; SPM, specialized pro-resolving mediators; TCR, T-cell receptor; TGF, transforming growth factor; TNF, tumor necrosis factor; VEGF, vascular endothelial growth factor. Question marks indicate absent or incomplete data and facts that have not been sufficiently studied for myocardial infarction.

However, results obtained in COLCOT and CANTOS clinical trials show the potential of anti-inflammatory therapy in AMI patients (150, 151), even though the benefits of approaches used up to date may seem not so large-scale and limited to the narrow group of patients. In our opinion relatively modest success of these trials is associated with a fact that inflammatory phase is obligatory at the early stage of MI to clear the necrotic cellular debris and create favorable circumstances for the development of reparative phase. Thereby, complete suppression of inflammation which may be beneficial during autoimmune conditions, may be inappropriate in patients after cardiovascular events at the early stages of treatment. New approaches for patient’s follow-up and treatment targeting resolution of inflammation should be elaborated.

**Figure 2:**
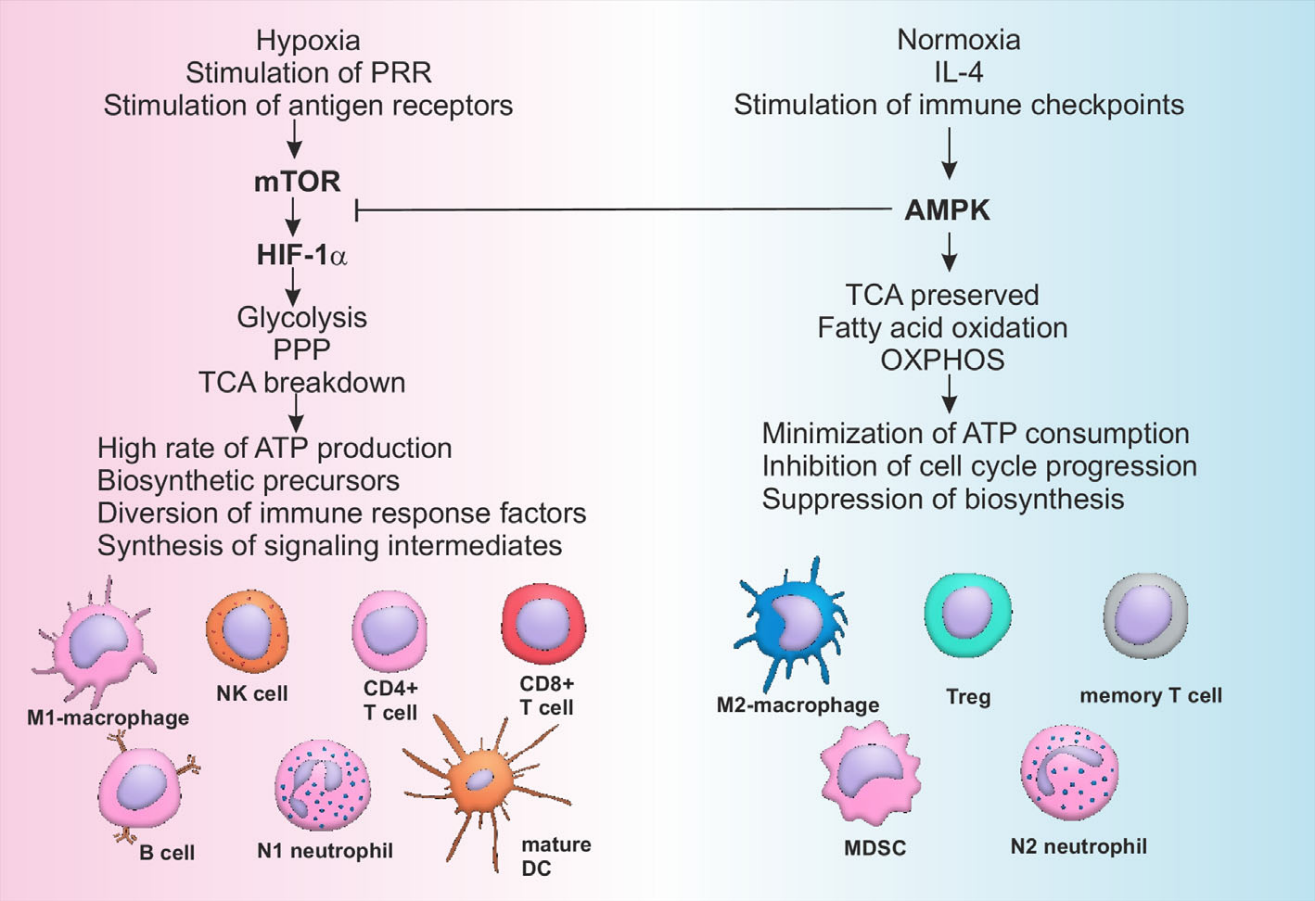
Pathways of metabolic reprogramming in immune cells. Activation of mTOR during hypoxia and stimulation of PRR by DAMPs or antigen receptors on T- and B-lymphocytes by antigens leads to the sustained stimulation of HIF-1α, which in turn activates glycolysis and pentose-phosphate pathway, breaking down tricarboxylic acid (TCA) cycle. All of the above mentioned metabolic pathways are associated with high rate of ATP production, generation of biosynthetic precursors (nucleotides, fatty acids, ribose, amino acids), diversion of immune suppressive factors such as phosphoenolpyruvate into glycolytic pathway and production of cofactors (such as NADH). As a consequence cells of the immune system are activated and acquire inflammatory phenotype. Restoration of the normal oxygen supply or anti-inflammatory signaling, sometimes combined with checkpoint (PD-1) activation, stimulates AMPK, characterized by the suppressive activity towards mTOR. AMPK supports integrity of TCA, stimulates OXPHOS and fatty acid oxidation, minimizing ATP consumption and inhibiting cell cycle and biosynthesis. Switching from catabolism to anabolism represents a stimulus for the development of anti-inflammatory and memory cells. AMPK, AMP activated protein kinase; ATP, Adenosine triphosphate; CD, Cluster of differentiation; DC, Dendritic cell; HIF, Hypoxia induced factor; IL, Interleukin; MDSC, Myeloid derived suppressor cell; mTOR, Mammalian target of rapamycin; OXPHOS, Oxidative phosphorylation; PPP, Pentose phosphate pathway; PRR, Pattern recognition receptors; TCA, Tricarboxylic acid cycle; Treg, T regulatory lymphocytes.

One of the modern concepts of immune modulation represents metabolic reprogramming of immune cells (152). All the inflammatory cells’ subpopulations have been shown to be dependent upon aerobic glycolysis, while anti-inflammatory subpopulations normally produce energy through oxidative phosphorylation (152). Rewiring of the cellular metabolism determines direction of cellular differentiation and may be used to selectively and specifically regulate the power of immune responses. Activation of AMP-activated protein kinase (AMPK) is known to increase fatty acid oxidation and promote differentiation of cells with pro-resolving activity (M2 macrophages, Tregs, N2 neutrophils), while stimulation of mammalian target of rapamycin (mTOR) and hypoxia induced factor (HIF) 1-α is associated with an increase of inflammatory profile of the immune cells (M1 macrophages, mature DCs, activated B cells, Th1, Th17) (**Figure 2**) (152–154).

Cellular metabolism is tightly linked to epigenetic reprogramming of the immune cells, which, in turn, represents another powerful tool of the cell fate control. Chromatin-modifying enzymes include multiple types of histone acetyltransferases and deacetylases. Moreover, some of these enzymes display non-catalytic activity, governing the direction of inflammatory responses. Recent work by Nguyen H.C.B. et al. demonstrated that histone deacetylase 3 (HDAC3) may activate either inflammatory or anti-inflammatory genes depending on the nature of the transcription factor being recruited (155).

The issue of targeted delivery of immune-modulating medications during MI also needs to be elaborated to avoid undesirable systemic effects. Current approaches propose to use hydrogels, patches, inhaled nano-particles, extracellular vesicles including exosomes and are reviewed in detail elsewhere (156). All the above mentioned techniques have certain dangers and drawbacks and require further studies of the bioavailability and safety in clinics.

New diagnostic approaches, such as fluorescence-based techniques, single-cell RNA sequencing and mass spectrometry imaging and profiling, which become more and more wide-spread and affordable, allow to evaluate single cell metabolomics and transcriptomics and propose personalized targeted ways of metabolic and epigenetic reprogramming depending on the initial state of immunometabolism (157, 158).

In the perspective it might be possible to find a powerful signaling mechanism to manipulate cellular heterogeneity in the course of the immune response during AMI and influence not only a single subpopulation, but redirect functional activity of the whole cellular diversity in a desired way. Identification of molecules that may be used during AMI to obtain time-dependent equilibrium in immune regulation through metabolic and epigenetic rearrangement represents a promising area of future research.

## Concluding Remarks

Increase of patients’ survival after AMI is associated with spread of HF frequency in population. Hence study of consequences of cellular response to myocardial injury will remain an important research area withholding both fundamental and practical significance. Summarizing our review, we can state that there are no “good” or “bad” players in the realm of the immune cells involved in myocardial remodeling after infarction. Each subset of immune cells present in the heart may either increase injury and inflammation or initiate healing of the cardiac tissue. Suppression of inflammation *via* total depletion of a definite cellular subset has not been associated with a more favorable outcome, or has led to inconsistent results. Rather, keeping a time-dependent balance in the work of immune cells, when inflammatory activity is followed by timely switching on of the resolution and reparative mechanisms, is what matters in the successful heart healing and remodeling following infarction.

The substantial question remains, which is the nature of the factors that orchestrate the preservation of the balanced immune response during myocardial infarction, and which are the factors that lead to the impairments in this process? We need to keep in mind, that patients face myocardial infarction already having a dramatic background of various health problems, including but not limited to atherosclerosis, other chronic inflammatory disorders, metabolic disturbances, increased blood pressure, aging, history of smoking and chronic stress, etc., all of which being associated with immune dysregulation of various kind and different scale. The amount of fundamental and clinical data obtained in the field up to date is enormous, but does not lead to the significant improvements. We need to admit that further breakthrough in management of post-infarction remodeling requires a novel instrument to systematize an exciting data. Possible solution may be the use of high-throughput methods and artificial intelligence to develop “digital counterparts” of inflammation which will allow creation of “inflammatory portrait” for each patient and identification of an existing gap in the work of immune cells in each single case. As a matter of fact, this foreshadows the end of the population-based medicine and beginning of the personalized medicine era.

## Author Contributions

VR and TS conceived the concept and designed the manuscript outline. IV and MS wrote the manuscript. IV prepared figures. TS and VR performed critical evaluation of the manuscript. IV prepared manuscript for publication. All authors contributed to the article and approved the submitted version.

## Funding

This work was supported by the Ministry of Science and Higher Education of the Russian Federation [theme of fundamental research AAAA-A15-115123110026-3].

## Conflict of Interest

The authors declare that the research was conducted in the absence of any commercial or financial relationships that could be construed as a potential conflict of interest.
